# The Ability of DNAJB6b to Suppress Amyloid Formation Depends on the Chaperone Aggregation State

**DOI:** 10.1021/acschemneuro.4c00120

**Published:** 2024-04-19

**Authors:** Andreas Carlsson, Emil Axell, Cecilia Emanuelsson, Ulf Olsson, Sara Linse

**Affiliations:** † Lund University, Biochemistry and Structural Biology, Lund, Naturvetarvägen 16, 223 62, Sweden; ‡ Lund University, Physical Chemistry, Lund, Naturvetarvägen 16, 223 62, Sweden

**Keywords:** Chaperone activity, Amyloid beta peptides, Amyloid inhibition, Self-assembly, Oligomer dissociation, Protein aggregation

## Abstract

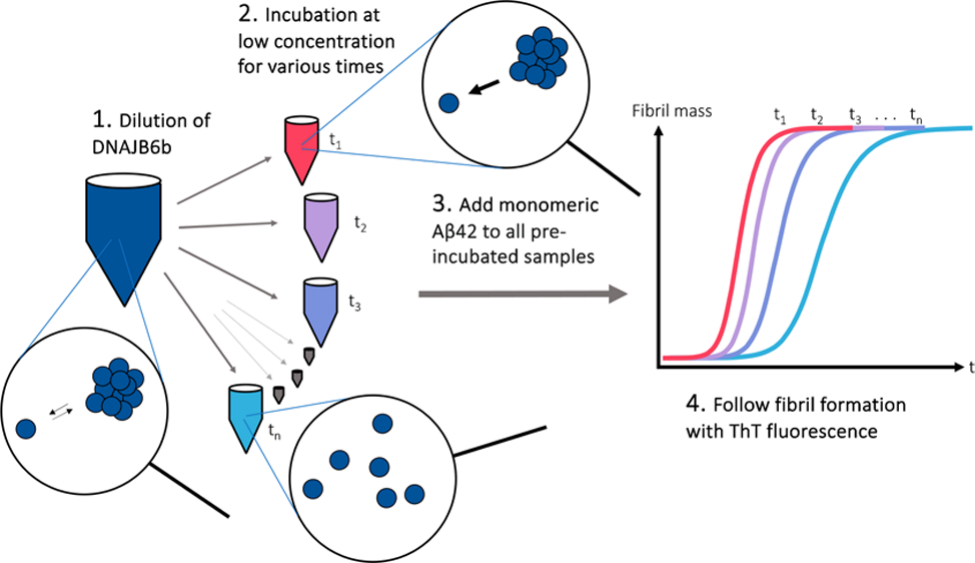

For many chaperones, a propensity to self-assemble correlates with function. The highly efficient amyloid suppressing chaperone DNAJB6b has been reported to oligomerize. A key question is whether the DNAJB6b self-assemblies or their subunits are active units in the suppression of amyloid formation. Here, we address this question using a nonmodified chaperone. We use the well-established aggregation kinetics of the amyloid β 42 peptide (Aβ42) as a readout of the amyloid suppression efficiency. The experimental setup relies on the slow dissociation of DNAJB6b assemblies upon dilution. We find that the dissociation of the chaperone assemblies correlates with its ability to suppress fibril formation. Thus, the data show that the subunits of DNAJB6b assemblies rather than the large oligomers are the active forms in amyloid suppression. Our results provide insights into how DNAJB6b operates as a chaperone and illustrate the importance of established assembly equilibria and dissociation rates for the design of kinetic experiments.

## Introduction

In many cases, chaperone self-assembly correlates with the activity. The highly efficient amyloid suppressing chaperone DNAJB6b (hereafter called JB6b) has a strong propensity to oligomerize.^1−4^ Which assembly states of JB6b are active in amyloid suppression remains to be established. Here, we show that the active form in the suppression of fibril formation of the amyloid β 42 peptide (Aβ42) is the JB6b subunit rather than the large oligomers. We use nonmodified JB6b to avoid issues regarding how mutations or labels might affect the chaperone activity. Note that we here use the concepts aggregation state and assembly state as synonyms to indicate the number of interacting proteins in one particle.

A common feature in protein aggregation diseases such as diabetes type II, Alzheimer’s, Parkinson’s, and Huntington’s diseases is the clustering of certain proteins and peptides into fibrillar structures, called amyloids. One way in which amyloidosis is endogenously suppressed is through the action of the type of proteins referred to as molecular chaperones^5^ and chaperone-like domains.^6^ Molecular chaperones, a few hundred proteins in humans, are conserved and maintain protein quality control, with age-related loss of function.^7,8^ Some chaperones operate together with other proteins using chemically stored energy such as ATP molecules, whereas others are potent amyloid suppressors by themselves.

The human chaperone JB6b belongs to both categories. It is efficient on its own in retarding amyloid formation and increasing the apparent solubility of amyloid prone peptides, called clients to the chaperone. JB6b is, like other J-domain proteins, involved in the HSP70 machinery, also including nucleotide exchange factors and ATP in the interactions with clients.^9−11^ JB6b is, both alone and as a cochaperone, an efficient suppressor of amyloid formation by several peptides/proteins, including amyloid β peptides,^1,12,13^ α-synuclein,^14−16^ polyglutamine peptides,^9,17−21^ IAPP,^22^ and TDP43.^23^ In addition to retardation of amyloid formation, JB6b has also been found to increase the apparent solubility of amyloid proteins.^13^ In the present work, we study the activity of JB6b *per se*, i.e., without any cochaperones, nucleotide exchange factors, or ATP. Previous investigations have established that the suppression of amyloid formation by JB6b is due to an interference with primary and secondary nucleation.^1,12^ This action has further been assigned to interactions of JB6b with aggregated, rather than monomeric, forms of the amyloid protein.^1,12^ However, it is not established whether the suppression requires oligomeric or subunit forms of JB6b. Hence, we investigate here which assembly states of JB6b are active in amyloid suppression.

It is not uncommon for chaperones to self-assemble into dimeric or higher order oligomeric structures, as reported for example in the case of DNAK,^24^ αB-Crystallin,^25^ and the DNAJB family of chaperons.^9^ JB6b has been reported to self-assemble into particles with a polydisperse size distribution, with reported sizes of about 7–24 nm in radius, depending on the protein concentration, solution conditions, and the accessible range of the measurement techniques.^1−4^ The self-assembly is concentration dependent, with an onset of oligomerization at around 120 nM JB6b, at room temperature, pH 8.0, and modest ionic strength. Below this concentration, JB6b has an average hydrodynamic radius of around 4–5 nm, possibly corresponding to dimers or a mixture of monomers, dimers, and other low number oligomers. Upper and lower limits of the hydrodynamic radii have been estimated to be 2.0–3.6 nm for monomeric JB6b, and 2.5–5.4 nm for dimeric JB6b.^4^ These smaller species will hereafter be called subunits of JB6b assemblies.

The role of the JB6b self-assembly is not yet clear. A mainly monomeric mutant of JB6b, called S/TΔ, has a highly reduced fibril suppression capacity,^13^ whereas cross-linked JB6b oligomers show no activity.^12^ There are examples of other chaperones which have been reported to dissociate into subunits to get full chaperone activity,^26−29^ and both BRICHOS and HSP60 are examples of amyloid suppressors that are more active when dissociated from larger oligomeric states.^30,31^

**Figure 1:**
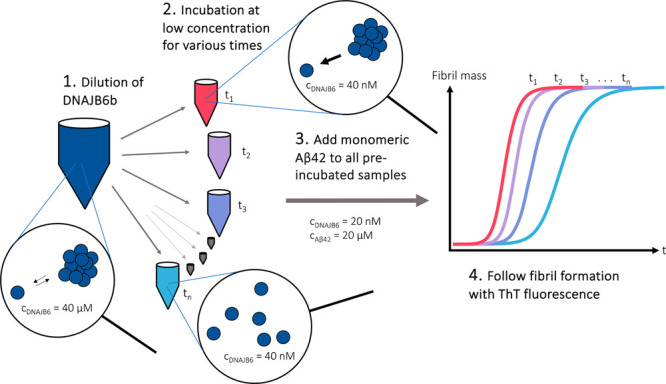
Schematic illustration of the experimental setup to investigate how the assembly state of JB6b affects its ability to suppress fibril formation of Aβ42. The experimental steps include (1) 1000-fold dilution of JB6b, (2) incubation at a low JB6b concentration for various times, (3) addition of Aβ42 monomers, (4) following the fibril formation via the thioflavin T (ThT) fluorescence intensity.

Here, we ask to what extent the assembly state of nonmodified JB6b affects amyloid formation. We thus investigate whether the large oligomers of JB6b suppress the fibril formation of Aβ42, or if it is mainly the chaperone subunits that are active amyloid suppressors. We examine this by monitoring the aggregation kinetics of Aβ42 in the absence or presence of a substoichiometric amount of JB6b (0.001:1 JB6b/Aβ42 molar ratio), where JB6b has been preincubated at a low concentration for various times after dilution (Figure 1).

## Results and Discussion

Figure 1 illustrates the experimental setup used to examine the role of the JB6b assembly state in its ability to retard Aβ42 fibril formation. JB6b at a high concentration, 40 μM, was diluted to 40 nM at various time points and loaded in a pegylated polystyrene 96-well plate in four replicates of 50 μL each. 40 nM is approximately three times lower than the estimated critical aggregation concentration (subunit solubility) of JB6b, and we expect at this concentration larger oligomers to dissociate into their subunits. The plate was kept at room temperature during successive loading to give incubation times of 200, 50, 25, 9, 3, 2, and 1 h and 30, 15, 8, 4, and 2 min, before 50 μL of 40 μM monomeric Aβ42 with 10 μM ThT was added. The resulting final concentrations were thus 20 nM JB6b, 20 μM Aβ42, and 5 μM ThT in 20 mM NaP, 0.2 mM EDTA, and pH 8.0. The amyloid formation was followed by monitoring the ThT fluorescence intensity at 37 °C under continuous reading with no shaking.

**Figure 2:**
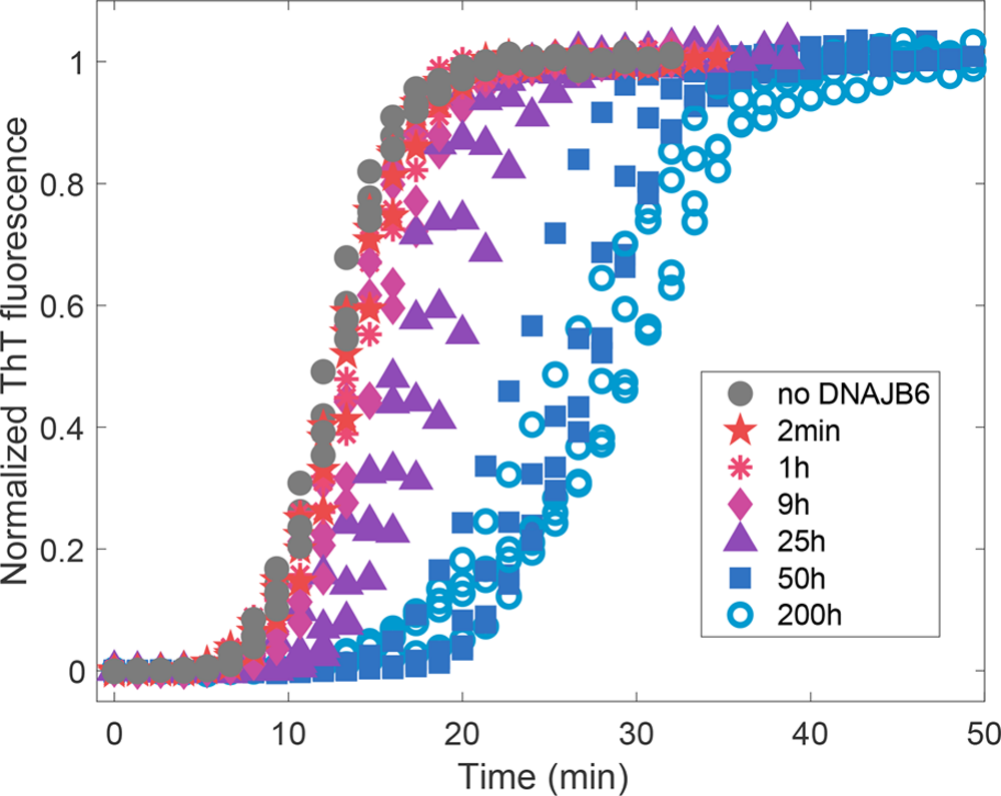
Aggregation kinetics of 20 μM Aβ42, displayed as the normalized fluorescence intensity of 5 μM ThT. A control without JB6b is shown in black filled circles. JB6b (20 nM) was added to the Aβ42 in all other samples (in colors), but the incubation time after JB6b dilution differs, to obtain samples with different assembly states of the chaperone. The incubation times are given as symbol and color descriptions in the figure, in replicates of four.

**Figure 3:**
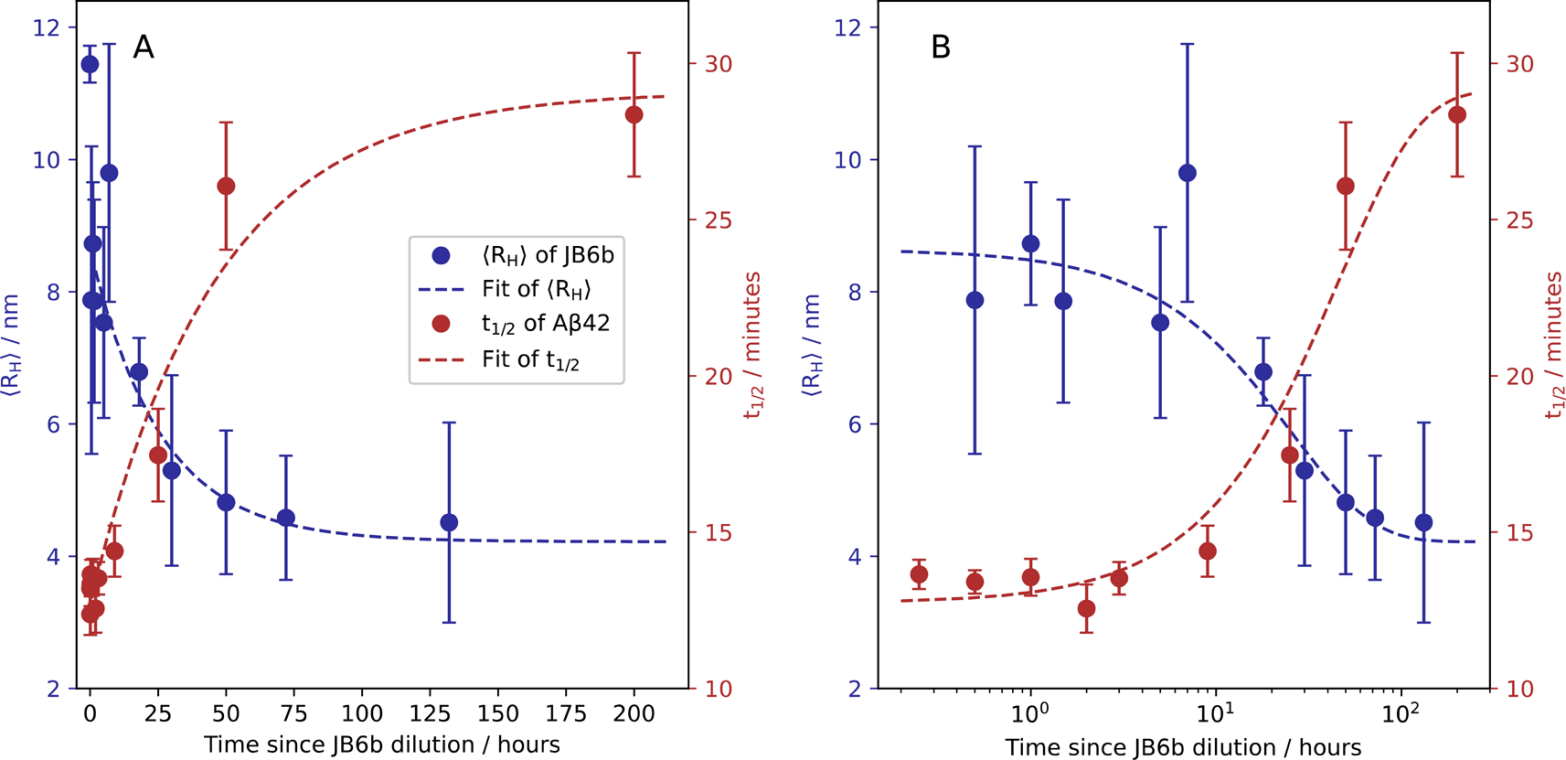
Comparison of the JB6b oligomer dissociation kinetics (blue, left *y* axis) and the Aβ42 fibril formation half time (red, right *y* axis), as a function of JB6b preincubation time. The error bars represent the standard deviations of four replicates for each sample. A fit to the fibril formation *t*_1/2_ data, in the form *t*_1/2_(*t*) = *A* – *B* e^–*k*_1_*t*^, is shown in dashed red, with the rate constant *k*_1_ = 0.022 h^–1^. In blue are replotted data from ref (4) of JB6b ⟨*R*_H_⟩, obtained using microfluidic diffusional sizing as a function of time after dilution from 6 μM to 100 nM. The fit is in the form ⟨*R*_H_⟩(*t*) = *D* e^–*k*_2_*t*^ + *F*, with an apparent dissociation rate constant *k*_2_ = 0.039 h^–1^ (dashed blue line). The data are displayed with linear and logarithmic time axes in panels A and B, respectively.

This procedure provided a situation in which Aβ42 aggregated in the absence or presence of chaperone at equal total concentration but with JB6b being in different assembly states depending on how much time had passed since its dilution. Examples of the resulting aggregation kinetics data are shown in Figure 2, with normalized fluorescence intensity as a function of time. When JB6b was diluted 2 min and 1 h before the Aβ42 addition, no or little effect on the aggregation kinetics was observed. First at 25 h or longer preincubation times, a clear suppression effect was observed. The kinetic traces were fitted using the established inhibition mechanism^1,12,13^ and the Amylofit^32^ online platform as described in Supporting Information, Figure S1. The same software was used to extract the halftime of aggregation, *t*_1/2_, for each kinetic trace, i.e., the time at which the fluorescence intensity has reached halfway between the initial baseline and final plateau (Figure 3).

The preincubation of JB6b was performed at room temperature to allow for comparison with earlier obtained dissociation kinetics, where 6 μM JB6b was diluted to 100 nM and the average hydrodynamic radius, ⟨*R*_*H*_⟩, was measured as a function of time, using microfluiodic diffusional sizing (MDS).^4^ The oligomer dissociation process seems to follow a single exponential decay with a rate that is concentration-independent when oligomers are diluted to below the subunit solubility (120 nM). Thus, a single exponential decay is used as a test function to fit the hydrodynamic radius decay as a function of time, ⟨*R*_H_⟩(*t*) = *A* – *B* e^–*k*_2_*t*^, where *k*_2_ is the apparent dissociation rate constant for which we obtain *k*_2_ = 0.039 h^–1^. The data of ref (4) can thus be used to analyze the amyloid suppression efficiency in light of which assembly states of JB6b are present. The JB6b dissociation data of ref (4) are replotted in Figure 3 (blue symbols), together with the *t*_1/2_ of Aβ42 fibril formation (red symbols), versus the pre-incubation times of JB6b at a low concentration (40 nM) before the addition of Aβ42. The dependence of fibril formation *t*_1/2_ with respect to preincubation time coincides well with the time evolution of the radius decrease, consistent with the subunits of JB6b being the active form in amyloid suppression.

The dissociation rate of JB6b assemblies upon dilution was, in the current work, studied using chemical cross-linking with BS3 (bis(sulfosuccinimidyl)suberate), and the cross-linked products were visualized in SDS-PAGE (data shown and discussed in Supporting Information, Figure S2). The data display a decline of cross-linked products with an apparent dissociation rate constant of 0.030 h^–1^, in agreement with MDS data and the effect on fibril formation.

The current results show that the large chaperone oligomers are essentially inactive and need to dissociate to their subunits to effectively suppress amyloid formation. One may ask why this phenomenon has escaped detection in earlier studies. The answer lies in the relative time scale of the events. The current findings were made possible by setting up the Aβ42 aggregation studies at such a high concentration (20 μM) that the lag time for aggregation is short relative to the dissociation time for the chaperone oligomers, while at the same time the chaperone is diluted so heavily at room temperature that the initial concentration of subunits is marginal. It should thus be noted that the difference in Aβ42 aggregation kinetics between having significantly dissociated JB6b and recently diluted JB6b is seen only when the lag phase of Aβ42 is much shorter than the dissociation time of JB6b and when JB6b is diluted from far above its subunit solubility. Most other studies have been conducted under conditions of a much longer Aβ42 lag phase, meaning that after the consumption of the initially present JB6b subunits into coaggregates with Aβ42, the remaining JB6b will have time to continuously dissociate to prolong the lag phase, which gives even more time for JB6b to dissociate etc. The resulting kinetics may in such a situation be similar to the case when JB6b was dissociated to start with. In line with this, Månsson et al.^1^ reported a similar Aβ42 aggregation kinetic when JB6b was added at different time points during the lag phase, compared to when an equal total amount was added initially.

Rather than being active as amyloid suppressors, the JB6b oligomers may be a consequence of the same chemical property that makes the subunits potent as suppressors. One such property may be a high chemical potential of the chaperone, which it could lower by forming self-assemblies or by forming coaggregates with amyloid peptides. The latter is one possible explanation for the fact that chaperones like JB6b not only delay amyloid formation but also enhance amyloid solubility.^13,33^ Future investigations may ask whether the molecular determinants of chaperone self-assembly and amyloid suppression are the same. One might also wonder whether there is any biological benefit to the large oligomers of JB6b, given their apparent inactivity. A speculation is that they act as an inactive and inert reservoir, providing a constant level of active JB6b in solution. The equilibrium distribution and exchange rates between the assembly states might be changed upon changes in cellular environment, as is for example reported for IbpP^26^ and DNAJA2.^27^ Indeed, another amyloid-suppressing chaperone, αB-Crystallin,^34^ has a high activation barrier and thus a strong temperature dependence of subunit dissociation.^35^

In conclusion, we have shown that the large DNAJB6b oligomers are not effective suppressors of Aβ42 fibril formation, but the chaperone is highly potent as a dissociated subunit. Our finding will likely extend to other amyloid forming peptides and likely also to other amyloid-suppressing chaperones.

## Methods

### Buffer and Chemicals

The buffer for all experiments was 20 mM sodium phosphate and 0.2 mM EDTA, at pH 8.0, filtered through a wwPTFE-filter (0.22 μm pore size) and degassed. All chemicals were of analytical grade. Thioflavin T (ThT) was purchased from CalBiochem.

### Protein Expression and Purification

JB6b and Aβ42 were expressed in *Escherichia coli* BL21 DE3 pLysS star and purified using a combination of sonication, centrifugation, ion exchange, and size exclusion steps as described.^36−38^ After the final SEC step, Aβ42 was lyophilized, whereas JB6b was frozen as a liquid. To prevent JB6b from precipitation during freezing, the protein was flash-frozen using a −80 °C precooled plastic block and tubes. Both proteins were stored at −20 °C. The JB6b amino acid sequence used was MVDYYEVLGVQRHASPEDIKKAYRKLALKWHPDKNPENKEEAERKFKQVAEAYEVLSDAKKRDIYDKYGKEGLNGGGGGGSHFDSPFEFGFTFRNPDDVFREFFGGRDPFSFDFFEDPFEDFFGNRRGPRGSRSRGTGSFFSAFSGFPSFGSGFSSFDTGFTSFGSLGHGGLTSFSSTSFGGSGMGNFKSISTSTKMVNGRKITTKRIVENGQERVEVEEDGQLKSLTINGKEQLLRLDNK.

The Aβ42 sequence (M1–42) was MDAEFRHDSGYEVHHQKLVFFAEDVGSNKGAIIGLMVGGVVIA.

### JB6b

An aliquot of the frozen 40 μM JB6b stock solution was thawed by placing the tube in a solid plastic rack at room temperature and diluted to 40 nM at the following time points before adding Aβ42: 200 h, 50 h, 25 h, 9 h, 3 h, 2 h, 1 h, 30 min, 15 min, 8 min, 4 min, and 2 min. Each JB6b sample was loaded in a costar 96-well half area plate (3881), in four replicates of 50 μL, with a tightly sealed cover to prevent the liquid from evaporating. The plate was left to incubate at room temperature (approximately 20 °C) until addition of Aβ42. Four wells were supplemented with 50 μL of buffer, i.e., no JB6b.

### Isolation of Aβ42 Monomers

Monomeric Aβ42 was isolated from a purified aliquot, dissolved in 1 mL of 6 M GuHCl, by SEC with a 10 × 300 mm Superdex75 column in freshly degassed buffer. The Aβ42 concentration was adjusted to 40 μM, based on the integrated absorbance peak at 280 nm in the chromatogram. ThT was added to a concentration of 10 μM from a 2 mM stock (prepared in water from powder, filtered through a wwPTFE-filter with a 0.22 μm pore size). The Aβ42 solution was kept on ice until use (ca. 1 h).

### Aggregation Kinetics Studies

200 h after the first dilution of JB6b and 2 min after the last one, 50 μL of isolated Aβ42 monomers were added to all wells, resulting in final concentrations of 20 μM Aβ42, 20 nM JB6b, and 5 μM ThT. The Aβ42 solution was added in the order from longest JB6b equilibration time to shortest, using a multichannel pipet to limit the total loading time to 1 min. The plate was immediately placed in a plate reader (FLUOstar Omega, BMG LABTECH) preincubated at 37 °C. The fibril mass concentration was probed by monitoring the ThT fluorescence intensity with excitation at 448 nm and emission at 480 nm. The data were collected without shaking, with stepwise reading with a reading cycle of 85 s and without pauses between reading cycles.

## Supporting Information Available

Kinetics fitting and the cross-linker study for examination of the DNAJB6b dissociation rate (PDF)

## Abbreviations

JB6b: DNAJB6b
ThT: thioflavin T
Aβ42: amyloid β 42 peptide
⟨*R*_H_⟩: average hydrodynamic radius
MDS: microfluidic diffusional sizing
