# Supplementary material for: The Ability of DNAJB6b to Suppress Amyloid Formation Depends on the Chaperone Aggregation State
Source: ACS Chem Neurosci. 2024 Apr 19;15(9):1732–7. doi: 10.1021/acschemneuro.4c00120 (PMC11066835; doi:10.1021/acschemneuro.4c00120)
Supplement: Supplementary file 1 — cn4c00120_si_001.pdf [file cn4c00120_si_001.pdf]

Supplementary information to:

# The Ability of DNAJB6b to Suppress Amyloid Formation Depends on the Chaperone Aggregation State

Andreas Carlsson[a*], Emil Axell[a], Cecilia Emanuelsson[a], Ulf Olsson[b] and Sara Linse[a]

[a]*Lund University, Biochemistry and Structural Biology, Lund, Naturvetarvägen 16, 223 62, Sweden.*
[b]*Lund University, Physical Chemistry, Lund, Naturvetarvägen 16, 223 62, Sweden.*

*Email: andreas.carlsson@biochemistry.lu.se

## Kinetic analysis

The data shown in Figure 2 were fitted using the Amylofit software [1] and a model including primary nucleation, secondary nucleation, and elongation. The coupled rate constants for primary nucleation and elongation ($k_n k_+$), and for secondary nucleation and elongation ($k_2 k_+$), were varied parameters, and the reaction orders for both primary and secondary nucleation were fixed at 2. The fits are displayed together with the kinetic data in Figure 1S. It was noted that some parameters can be globally fitted and still give a good representation of the data, but the current experiment was not designed to evaluate the inhibitory mechanism of JB6b, which has been explored before [2,3].

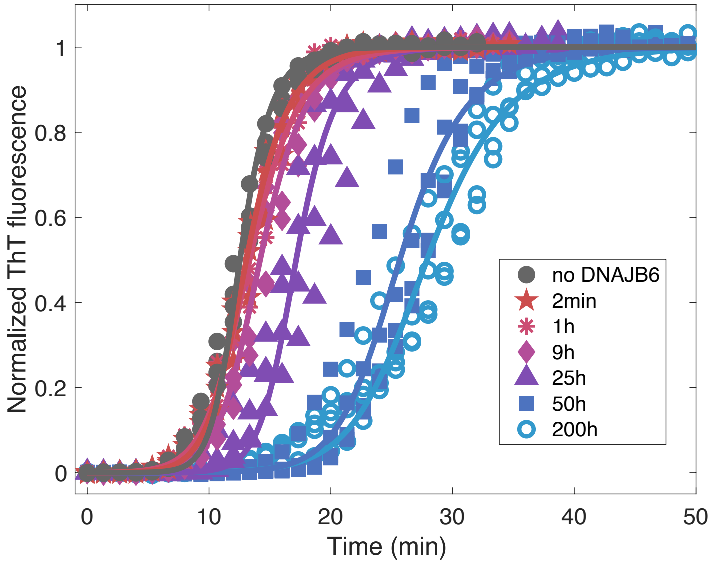


**Figure S1**. Aβ42 aggregation kinetics with fitted kinetics from Amylofit, where the coupled rate constants $k_n k_+$ and $k_n k_2$ were varied parameters.

## Crosslinking of 40 nM JB6b

To monitor the dissociation kinetics of JB6b oligomers upon dilution with a complementary technique to MDS, chemical crosslinking was utilized. The experimental setup is illustrated in Figure S2, panel A. 12.8 µM JB6b was diluted to 40 nM in a volume of 100 ml. Fractions of 5 ml were removed from the container as a function of time since dilution, and crosslinked using either 40 µM (Figure S2, panel B) or 400 µM (Figure S2, panel C) BS3 linker (bis(sulfosuccinimidyl)suberate). The BS3 linker was prepared by dissolving in $H_2O$ to 40 mM just prior to freezing in 5 µl aliquots in 5 ml tubes (Eppendorf, protein low binding). Each tube was thawed less than a minute before the addition of 5 ml protein solution. The reaction was stopped after 10 minutes by adding 40 mM ethanolamine from a 1 M stock, pH 8.0. The samples were concentrated by precipitation by the addition of 10 % (v/v) tri-chloro acetic acid (TCA), vortexing, incubation on ice for a minimum of 30 min, centrifugation at 10 000 g at 4 °C for 20 min and pellet resuspension in 3 µl of 3 M Tris at pH 8.0. 3 µl of 4X SDS loading buffer was added to each sample and SDS-PAGE was run at 140 V for 110 min using 10-20% polyacrylamide Tris/Tricine gels (Novex).

One should note that oligomers can be partly crosslinked. Under denaturing conditions such as in SDS-PAGE, the assemblies will dissociate into the smallest crosslinked species, which may result in bands corresponding to smaller sizes in an SDS-PAGE than the complexes they were part of under the native solution condition. Partial crosslinking is a probable explanation for the results shown in Figure S2 panel B (40 µM crosslinker). Here, mostly monomers and low order oligomers are visible at the shortest times after dilution (for instance in lane 2, 2 min after dilution). When using 10 times higher concentration of crosslinker, Figure S2 panel C, much less monomers and low order oligomers are seen shortly after dilution, but instead a large part of the protein is retained high up in the lane, not penetrating the gel more than a few millimeters. Hence, in the case of 400 µM crosslinker, the large JB6b oligomers are crosslinked.

To quantify the results of the gel in Figure S2, panel B, the software ImageJ was used to get grey value profiles of each lane. An example of how this analysis was carried out for lane 2 is shown in Figure S2, where the analyzed part is the drawn rectangle in panel B, and the generated profile is shown in panel D. The integral of each protein band was calculated to a baseline between two chosen points on the profile. Due to the many experimental steps that leads to the protein loaded in each lane, not the same amount of protein was able to be loaded for each sample. Hence, to compare the amount of crosslinked oligomeric states between lanes, the sum of the non-monomeric integrals was divided by the sum of all integrals in that lane. The ratio is plotted versus the time since dilution in panel E, in black circles. A fit to an exponential decay is shown as dashed line, where the apparent dissociation time constant is $\mathrm{k}_3 = 0.030$ h$^{-1}$. The control sample without any added crosslinker is plotted at time zero as a blue square and is not part of the fitted curve. The dissociation time constant is in the same size range as obtained using MDS in Ref. 4 ($k_2 = 0.039$ h$^{-1}$). It should be noted that the $\langle R_H \rangle$ from MDS not necessarily relates perfectly to the amount of crosslinked oligomeric species, since the crosslinking only reports on a part of the self-associated protein. In other words, since we do not know if some aggregation states are more or less prone to be crosslinked, the decay rate does not need to follow the $\langle R_H \rangle$ decay precisely, but should at least be on the same

order of magnitude, which they are. Furthermore, in both measurement techniques there is a rather large measurement error.

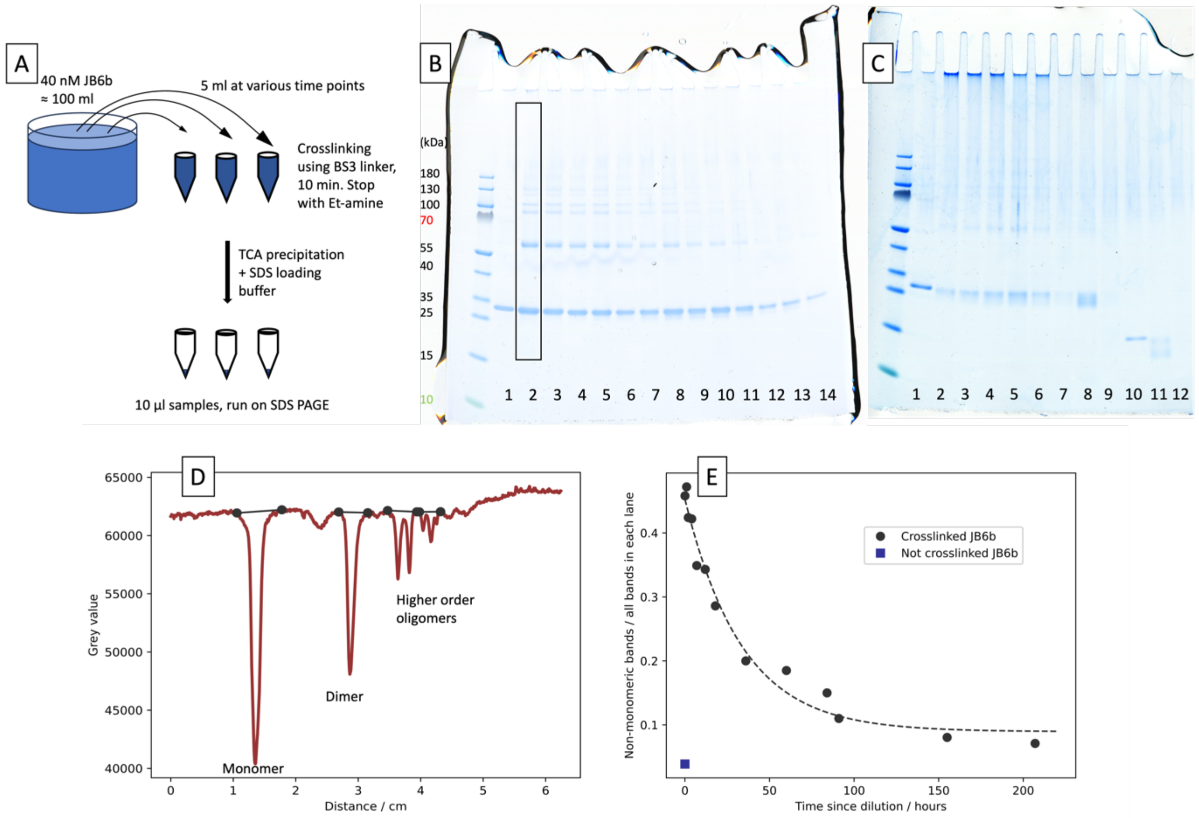


***Figure S2.*** *SDS-PAGE and gel analysis of JB6b at 40 nM, concentrated using TCA precipitation and stained after electrophoresis with Instant Blue to be visualized on a gel.* ***Panel A****: illustration of the experimental workflow.* ***Panel B:*** *scanned gel where 40 µM of BS3 crosslinker was used. Lane 1: only JB6b, no crosslinker added. Lane 2-14: JB6b equilibrated at room temp for the following times after dilution (lane number): 2 min (2), 1 h (3), 2 h (4), 4 h (5), 7 h (6), 12 h (7), 18 h (8), 36 h (9), 60 h (10), 84 h (11), 91 h (12), 155 h (13), 207 (14). The rectangle shows the area used in the image analysis in panel D, in the example case of lane 2 (2 min equilibration time).* ***Panel C:*** *scanned gel where 400 µM crosslinker was used. Lane 1: only JB6b, no crosslinker added. Lane 2-9: JB6b equilibrated at room temp for the following times after dilution (lane number): 2 min (2), 1 h (3), 3.5 h (4), 7 h (5), 27 h (6), 75 h (7), 210 h (8), 360 h (9). Lane 10: diS100G with no crosslinker. Lane 11: diS100G with 400 µM crosslinker. Lane 12: empty.* ***Panel D****: an example of a grey value profile, generated using ImageJ "Plot profile" of lane 2 in panel B (the drawn rectangle). 0 cm corresponds to the top part of the rectangle. The baselines are shown which were used for calculating integrals of the bands corresponding to monomers, dimers, and higher order oligomers.* ***Panel E:*** *the integral of all non-monomeric bands normalized to the total amount of stained protein in each lane (non-monomeric band integral / (non-monomeric band integral + monomeric band integral), plotted versus the equilibration time of JB6b (black circles). The dashed line is a fit to the data of the crosslinked samples, using a single exponential decay,* $f(t) = Ge^{-k_3 t} + H$ *, where* $k_3$*, G and H are fitted constants, with the time constant* $k_3 = 0.030$ *$h^{-1}$ The blue square is a sample with the same treatment as the other, but without any crosslinker added.*

One might ask whether a crosslinked product could be the result of two particles coming in proximity by Brownian motion and get crosslinked at this moment, providing an overestimation of the fraction of oligomeric species. This hypothesis was here discarded using a monomeric control protein, with similar number of reactive sites for the crosslinker. In this case, a protein consisting of two copies of human S100G, joined via a single glycine residue, was expressed as a single protein chain, and used as a control. The amino acid sequence for diS100G was:
MKSPEELKRIFEKYAAKEGDPDQLSKDELKLLIQAEFPSLLKGMSTLDDLFQELDKDGDGEVSFEEFQVLVK KISQGRSPEELKRIFEKYAAKEGDPDQLSKDELKLLIQAEFPSLLKGMSTLDDLFQELDKDGDGEVSFEEFQV LVKKISQ.

This designed protein is about 20 kDa in Mw and has 19 lysine residues and one N-terminus, thus an equal number of primary amines, i.e. reactive sites for BS3 crosslinker, as JB6b. Given the highly inert nature of S100G, it was expected to appear monomeric, which is also the case as seen in Figure S2 panel B, lane 10 (no crosslinking) and lane 11 (crosslinked using 400 µM BS3 crosslinker). In the crosslinked case, internal crosslinks prevent full denaturation under the conditions of the SDS PAGE, making some of the crosslinked protein appear smaller than the non-crosslinked protein. But importantly, no traces of low order oligomers can be seen, suggesting that crosslinking due to random proximity by Brownian motion is negligible.

# References


1. Meisl, G. *et al.* Molecular mechanisms of protein aggregation from global fitting of kinetic models. *Nat Protoc* **11**, 252–272 (2016).
2. Månsson, C. *et al.* Interaction of the molecular chaperone DNAJB6 with growing amyloid-beta 42 (Aβ42) aggregates leads to sub-stoichiometric inhibition of amyloid formation. *Journal of Biological Chemistry* **289**, 31066–31076 (2014).
3. Månsson, C. *et al.* Conserved S/T Residues of the Human Chaperone DNAJB6 Are Required for Effective Inhibition of Aβ42 Amyloid Fibril Formation. *Biochemistry* **57**, 4891–4892 (2018).
4. Carlsson, A., Olsson, U. & Linse, S. On the micelle formation of DNAJB6b. *QRB Discov* **4**, (2023).
